# Dual RNA-seq reveals no plastic transcriptional response of the coccidian parasite *Eimeria falciformis* to host immune defenses

**DOI:** 10.1186/s12864-017-4095-6

**Published:** 2017-09-05

**Authors:** Totta Ehret, Simone Spork, Christoph Dieterich, Richard Lucius, Emanuel Heitlinger

**Affiliations:** 1 Institute of Biology, Molecular Parasitology, Humboldt-Universität zu Berlin, Philippstr. 13, Haus 14, 10115 Berlin, Germany; 2 FG16 - Mycotic and parasitic agents and mycobacteria, Robert Koch Institute, Berlin, Germany; 3 University Hospital Heidelberg - German Center for Cardiovascular Research (DZHK), Analysezentrum III, Im Neuenheimer Feld 669, 69120 Heidelberg, Germany; 4 Leibniz Institute for Zoo and Wildlife Research, Research Group Ecology and Evolution of Parasite Host Interactions, Alfred-Kowalke-Str. 17, 10315 Berlin, Germany

**Keywords:** Phenotypic plasticity, Parasite lifecycle, Transcriptional plasticity, Apicomplexa, Dual RNA-seq, Dual transcriptomics, Coccidia

## Abstract

**Background:**

Parasites can either respond to differences in immune defenses that exist between individual hosts plastically or, alternatively, follow a genetically canalized (“hard wired”) program of infection. Assuming that large-scale functional plasticity would be discernible in the parasite transcriptome we have performed a dual RNA-seq study of the lifecycle of *Eimeria falciformis* using infected mice with different immune status as models for coccidian infections.

**Results:**

We compared parasite and host transcriptomes (dual transcriptome) between naïve and challenge infected mice, as well as between immune competent and immune deficient ones. Mice with different immune competence show transcriptional differences as well as differences in parasite reproduction (oocyst shedding). Broad gene categories represented by differently abundant host genes indicate enrichments for immune reaction and tissue repair functions. More specifically, TGF-beta, EGF, TNF and IL-1 and IL-6 are examples of functional annotations represented differently depending on host immune status. Much in contrast, parasite transcriptomes were neither different between Coccidia isolated from immune competent and immune deficient mice, nor between those harvested from naïve and challenge infected mice. Instead, parasite transcriptomes have distinct profiles early and late in infection, characterized largely by biosynthesis or motility associated functional gene groups, respectively. Extracellular sporozoite and oocyst stages showed distinct transcriptional profiles and sporozoite transcriptomes were found enriched for species specific genes and likely pathogenicity factors.

**Conclusion:**

We propose that the niche and host-specific parasite *E. falciformis* uses a genetically canalized program of infection. This program is likely fixed in an evolutionary process rather than employing phenotypic plasticity to interact with its host. This in turn might limit the potential of the parasite to adapt to new host species or niches, forcing it to coevolve with its host.

**Electronic supplementary material:**

The online version of this article (10.1186/s12864-017-4095-6) contains supplementary material, which is available to authorized users.

## Background

The term plasticity describes the ability of genetically identical organisms to display variable phenotypes, e.g., via different developmental or metabolic programs. So called reaction norms govern how a particular genotype is translated into a phenotype depending on environmental stimuli [1]. The presence of predators is known to alter, e.g., developmental programs of genetically identical prey animals to produce different phenotypes (reviewed in [2]). Infections by pathogens are known to alter host phenotypes: in fact all non-constitutive immune reactions can be regarded as a manifestation of plasticity [3]. Hence, to understand the outcomes of parasitic infections and host-parasite interactions the concept of plasticity is useful.

The reciprocal effect of the within-host environment on parasite phenotypes, i.e. plasticity, is less studied, especially in parasites of animals. For many parasite species it remains unclear whether differences in pathology are due to parasites’ genotypic or phenotypic (plastic) differences, the latter resulting from host-parasite interactions, e.g., host immune responses. An exception are Nematode infections (reviewed by [4]), in which for example worm length [5] and other aspects of morphology [6], or developmental timing [7] has been shown to vary with host genotype. However, it is unclear to which extent such differences a) are passively imposed on the parasite, or b) an adaptive response of the parasite. Such adaptive plasticity might be a determinant of the extent of host specialisation, the likelihood of host-switches and eliminately the degree to which co-speciation and co-adaptation (together defining co-evolution) are observed.

*E. falciformis* is an intracellular parasite in the phylum Apicomplexa, which comprises more than 4000 described species [8]. Prominent pathogens of humans are found in this phylum, such as *Toxoplasma gondii*, the causative agent of toxoplasmosis, *Plasmodium* spp., causing malaria, and *Cryptosporidium* spp., which cause cryptosporidosis. Coccidiosis is a disease of livestock and wildlife caused by coccidian parasites which are dominated by >1800 species of *Eimeria* [8]. The genus is best known for several species which are problematic for the poultry industry [9]. *E. falciformis* naturally infects wild and laboratory *Mus musculus*, and its genome is sequenced and annotated making it a useful model for studying *Eimeria* spp. [10]. The parasite has its niche in the cecum and upper part of colon, mainly in the cells of the crypts [11, 12]. This monoxenous parasite goes through asexual (schizogony) and sexual reproduction, which results in the host releasing high numbers of oocysts approximately between day six and 14 after infection. When a mouse ingests *E. falciformis* oocysts, one sporulated oocyst releases eight infective sporozoites inside the host, which infect epithelial crypt cells. Within the epithelium, merozoite stages form in several rounds of asexual reproduction, followed by gamete formation and sexual reproduction, within the same host. Schizogony takes place approximately until day six and then gametes form and sexual reproduction takes place, resulting in unsporulated oocyst shedding. Schizogony is not completely synchronous; the exact number of schizogony cycles is unclear and could vary naturally [11, 13]. There is evidence for a genetic predisposition of *Eimeria* spp. to perform different numbers of schizogony cycles, as parasites can be selected to become “precocious”, completing the lifecycle faster with a reduced number of schizogony cycles [14, 15]. Additionally, it has been shown that *Eimeria vermiformis*, also a parasite of *M. musculus* intestines, displays prolonged patency (period of oocyst shedding) but an unaltered length of pre-patency periods in mice of different immmune status [16–18]. Whether this developmental plasticity in *E. vermiformis* is reflected on the transcriptional level of that parasite has not been investigated. Timing of both patency and pre-patency was shown to be non-plastic in *E. falciformis* var. pragensis [17]. Beyond developmental timing it is not known whether parasite strategies – i.e. processes optimizing host exploitation – are plastic and can be triggered by exogenous stimuli, such as host immune responses.

*Eimeria* spp. generally induce host protection against reinfection [19–25] and T-cells play a major role [21, 23, 24]. In response to *E. falciformis* infection of laboratory mice, interferon gamma (IFNγ) is upregulated [12, 26]. In an IFNγ-deficient mouse host model which displays larger weight losses and intestinal pathology but also lower oocyst output for *E. falciformis*, the wild-type phenotype was recovered by blocking IL-17A and IL-22 signaling [26]. Also in *E. verminformis* IFNγ, interleukin-6 (IL-6), and major histocompatibility complex (MHC) class I and II have been shown to be required for protective immune reactions in mice [25]. These studies demonstrate that adaptive immunity clearly plays a role in limiting the reproductive success of *Eimeria* spp. infection, but effects on the parasite, apart from reproductive output, remain poorly understood. It is an open question whether the parasite is passively impacted or responds, e.g., via changes in its transcriptome, to changes in the host immune response.

We used a “dual RNA-seq” approach, i.e., we simultaneously assessed the transcriptomes of host and parasite in biological samples containing both species [27–31]. Applying this to an infection of *E. falciformis* in the mouse, we produced host and parasite transcriptomes from the same samples, tissue, and time-points. We describe and analyze host and parasite mRNA profiles at several time-points post infection and contrast transcriptomes of naïve and challenge-infected wild-type mice to hosts with strong deficiency in adaptive immune responses. This approach allows us to screen transcriptional changes which may be involved in host-parasite interactions for plasticity to alterations in the host immune system. We hypothesize that changes in the parasite transcriptome would be indicative of a plastic response allowing for functionally altered infection programs.

## Results & discussion

### Immune competent hosts induce protective immunity against *E. falciformis* infection

To investigate *E. falciformis* development throughout the lifecycle in a natural mouse host (NMRI mice) dual transcriptomes were produced at 3, 5, and 7 days post infection (dpi), which are suitable time points to assess asexual and sexual developmental stages of the parasite in its host [11, 13]. We also investigated parasite development and transcriptomes in a mouse strain which is severely limited in adaptive immune responses (*Rag1* ^*−/−*^; “immunocompromised” hereafter) with *Rag1* ^*−/−*^ and the respective isogenic background strain (C57BL/6 as control) at day 5 post infection. To further elucidate host immune responses and parasite sensitivity to host immunity, we also challenge infected all mouse groups (i.e. infected after recovery of a first infection; see Methods) and sampled at the same time-points as in naïve mice.

**Fig. 1:**
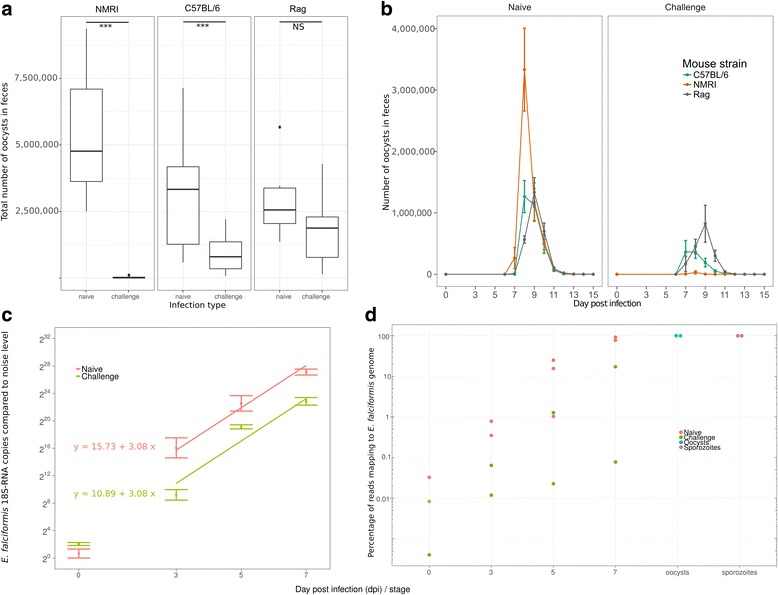
Oocyst output and changes in intensity of *E. falciformis* infection in mouse. Oocyst counts in naïve and challenge infection are shown for three different mouse strains. For infection of naïve NMRI 150 oocysts were used, for challenge infection 1500 oocysts. For C57BL/6 and *Rag1* ^*−/−*^ mice 10 oocysts were used in each infection. **a** Overall output of shed oocysts and (**b**) shedding kinetics are depicted. **c** RT-qPCR data of *E. falciformis* 18S in NMRI mice displays an increase in parasite mRNA over the course of infection. Significantly less parasite 18S transcripts (normalized against host transcripts of house-keeping genes) were detected in challenge infected mice. Formulas and prediction lines are given for linear models. **d** The percentage of parasite mRNA detected by RNA-seq increases during infection (shown for NMRI). More mRNA is detected in naïve mice compared to challenge infected mice. Sporozoites and oocysts contained ~100% parasite material

Infections showed drastically decreased oocyst output (Fig. 1a and b) in immune competent hosts undergoing a second, challenge infection compared to naïve animals infected for the first time (Mann–Whitney test, in NMRI, *n* = 12, U = 32, *p* = 0.004; in C57BL/6, *n* = 24, U = 111, *p* = 0.008). Similarly, a strong reduction of parasite 18S rRNA in the challenge infection down to 3.5% of the amount measured in naïve hosts was detected in reverse transcription quantitative PCR (RT-qPCR) in NMRI hosts (Fig. 1c). The model inferring this had a good fit (R^2^ = 0.94) and the change of the intercept for challenged compared to naïve hosts was highly significant (*t* = −6.71; *p* < 0.001). Differences in the slope were not significant (*t* = −1.522; *p* = 0.15), indicating that the amount of parasite material on 3 dpi is sufficient to explain a linear increase until 7 dpi. Overall this data is in line with the strong reduction of oocyst shedding seen in challenge infected immune competent mice, but also suggests that the host immune defense disturbs the parasite already at an early stage of infection, possibly even before 3 dpi.

In contrast, in immune deficient mice no significant difference in parasite reproductive success (Fig. 1a) was observed between naïve and challenge infection (Mann–Whitney test; *n* = 24, U = 96, *p* = 0.10). Both in the immunocompromised and immune competent animals, however, all mice had cleared the infection by day 14. We thereby note that *E. falciformis* infection is self-limiting also in mice without mature T- and B-cells, however with a delayed peak of oocyst shedding in immune deficient hosts (Fig. 1b). *E. vermiformis*, in contrast*,* has been shown to display prolognged patency (shedding of oocysts up to 23 instead of 16 dpi) in immunocompromised hosts [16–18]. In comparison, the delayed peak of shedding we observe for *E. falciformis* in immunocompromised hosts does not affect pre-patency and patency periods (beginning and end of oocyst shedding), confirming earlier reports of largely lacking developmental plasticity in *E. falciformis* [17]. We take advantage of the presence of the same lifecycle stages in hosts of varying immune competence to assess whether E. falciformis optimizes its host exploitation strategies in response to varying host defenses.

### Parasite and host dual transcriptomes can be assessed in parallel

We found the increase in parasite numbers over time after infection to also be reflected by the proportion of *E. falciformis* mRNAs sequenced in the combined pool of transcripts from host and parasite (for NRMI mice in Fig. 1d). Using mRNA from infected cecum epithelium we demonstrate that even early in infection (3 dpi, during early asexual reproduction) there is sufficient parasite material to detect parasite mRNAs in the pool including host mRNAs, and to quantify individual host and parasite mRNA abundance (Table 1). The number of total (host + parasite) read mappings for individual replicates ranged from 25,362,739 (sample Rag_1stInf_0dpi _rep1) to 230,773,955 (NMRI_2ndInf_5dpi_rep1). Similar to qPCR, a minimal level of background noise in the abundance estimates of *E. falciformis* transcripts is detected in RNA-seq for uninfected mice at 0 dpi.

**Table 1:** Summary of data per sample, sorted according to number of reads mapping to the *E. falciformis* genome

Sample^a^	Sequencing method	Batch	Total reads	Reads mapping mouse	Reads mapping *E. falciformis*	Percentage *E. falciformis*	# *E. falciformis* genes^b^
NMRI_2ndInf_0dpi_rep1	GAII	2	108,937,797	70,489,674	247	0.0004	1
Rag_1stInf_0dpi_rep1	hiseq	3	25,362,793	18,853,850	443	0.0023	2
C57BL/6_1stInf_0dpi_rep1	hiseq	3	35,731,249	25,119,348	457	0.0018	2
C57BL/6_1stInf_0dpi_rep2	hiseq	3	47,085,959	34,377,133	608	0.0018	2
Rag_1stInf_0dpi_rep2	hiseq	3	46,556,156	35,233,327	676	0.0019	2
NMRI_2ndInf_0dpi_rep2	hiseq	3	58,122,244	40,794,245	3406	0.0083	51
NMRI_2ndInf_3dpi_rep1^c^	hiseq	3	57,934,016	40,544,287	4803	0.0118	95
NMRI_2ndInf_5dpi_rep2^c^	hiseq	3	63,965,539	48,289,181	10,941	0.0227	407
NMRI_1stInf_0dpi_rep1^c^	GAII	1	82,364,585	55,176,243	17,954	0.0325	701
NMRI_2ndInf_3dpi_rep2	hiseq	3	65,548,826	46,171,909	29,548	0.0640	1580
NMRI_2ndInf_7dpi_rep2	hiseq	3	67,487,466	51,722,265	40,091	0.0775	1836
Rag_1stInf_5dpi_rep1	hiseq	3	38,651,359	29,982,453	63,024	0.2098	2548
Rag_1stInf_5dpi_rep2	hiseq	3	34,779,832	25,297,803	99,000	0.3898	2828
C57BL/6_1stInf_5dpi_rep1	hiseq	3	40,904,388	29,319,604	185,969	0.6303	4173
Rag_2ndInf_5dpi_rep1	hiseq	3	50,049,848	37,093,621	192,856	0.5172	4167
C57BL/6_1stInf_5dpi_rep2	hiseq	3	29,511,368	18,062,349	215,696	1.1801	3823
C57BL/6_2ndInf_5dpi_rep1	hiseq	3	35,148,432	25,660,184	262,909	1.0142	4563
NMRI_1stInf_3dpi_rep1	GAII	1	73,236,430	49,993,358	394,384	0.7827	5220
NMRI_1stInf_3dpi_rep2	GAII	2	160,709,694	117,791,044	413,051	0.3494	4862
NMRI_1stInf_5dpi_rep2	GAII	2	119,902,722	76,419,774	794,570	1.0290	5333
NMRI_2ndInf_5dpi_rep1	GAII	2	230,773,955	143,186,486	1,846,840	1.2734	5533
NMRI_2ndInf_7dpi_rep1	hiseq	3	70,366,762	41,467,146	8,634,201	17.2335	5875
NMRI_1stInf_5dpi_rep1	GAII	2	76,702,168	47,037,087	8,669,701	15.5631	5700
Sporozoites_rep2	GAII	0	19,551,681	8656	11,470,604	99.9246	5513
NMRI_1stInf_5dpi_rep3	GAII	0	191,099,180	83,735,624	27,839,458	24.9513	5784
NMRI_1stInf_7dpi_rep1	GAII	1	66,505,514	3,310,666	39,400,884	92.2488	5932
Sporozoites_rep1	GAII	1	67,325,397	4334	43,774,401	99.9901	5825
Oocysts_rep1	GAII	1	68,859,802	3805	49,653,065	99.9923	5695
Oocysts_rep2	GAII	0	151,090,783	18,524	71,019,860	99.9739	5777
NMRI_1stInf_7dpi_rep2	GAII	1	139,749,046	21,699,324	73,539,445	77.2159	5943

^a^Sample names are given with information separated by underscore as follows: 1) mouse strain, 2) naïve (1^st^) or challenge (2^nd^) infection, 3) dpi (days post infection), and 4) replicate number

^b^Number of expressed *E. falciformis* genes (read counts >5)

^c^These samples were removed from downstream analyses because of uncertain infection status

We did not detect bias in overall mRNA abundance patterns induced by, e.g., use of different sequencing platforms (and resulting differences in overall depth of sequencing), or by groups of samples processed in parallel (experimental batches) using a multivariate technique (multidimensional scaling, MDS; Additional file 1: Figure S1). Efficient normalization was confirmed in that samples with large differences in parasite read proportions show similar transcriptome signatures. This normalization also resulted in unimodal distributions of read numbers (Additional file 2: Figure S2) in agreement with negative binomial distributions assumed for statistical modeling and testing.

Remarkably, at 7 dpi before oocyst shedding peaks, samples from infected naïve mouse epithelium contained 77% and 92% parasite mRNA, i.e., drastically more mRNA from the parasite than from the host (Fig. 1d and Table 1). Our transcriptomes for these late infection samples are in agreement with previously published microarray data from mice infected with *E. falciformis* [12], as log2 fold-changes at our 7 dpi versus controls correlated strongly – for given mRNAs – with log2 fold changes at 6 dpi versus controls in that study (Spearman’s σ = 0.72, *n* = 9017, *p* < 0.001; Additional file 3: Figure S3). Considering both biological differences in the experiments, such as exact time-points for sampling, and technical differences between the two methods, this correlation confirms the adequacy of using dual RNA-seq for assessing the host transcriptome in the presence of large proportions of parasite mRNA. Below, we first describe changes in the mouse transcriptome and suggest possible mechanisms at play. Variance in host transcriptome changes upon infection constitutes a potential environmental stimulus for parasites to react on, as addressed later.

### The mouse transcriptome undergoes large changes upon *E. falciformis* infection

**Fig. 2:**
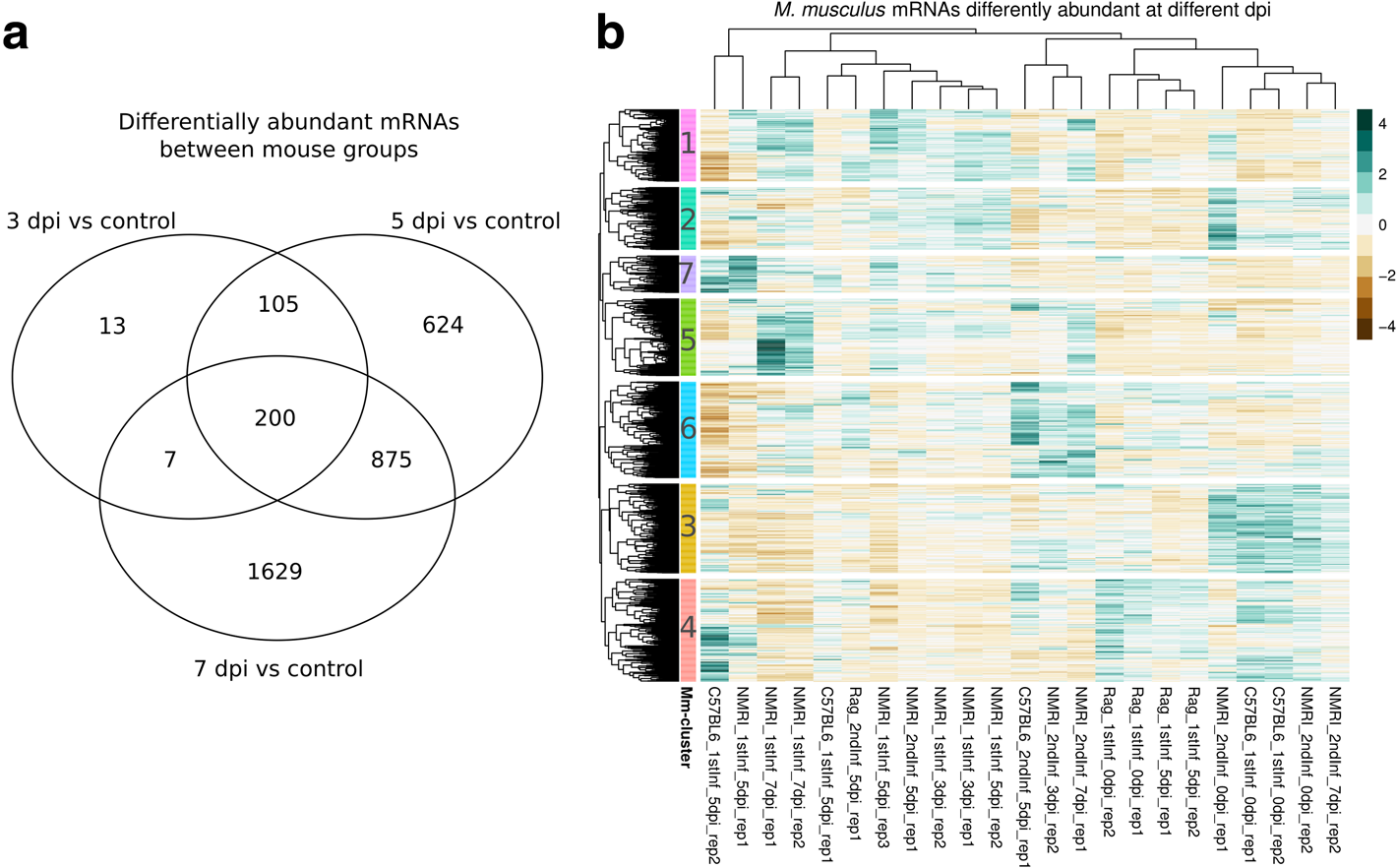
Differentially abundant mouse mRNAs and clustering thereof. **a** Venn diagram visualizes the overlap between genes showing differential abundance (FDR < 0.01; edgeR glm likelihood-ratio tests) between i) uninfected controls and different time-points post infection and ii) between different time-points and the sum of all genes reacting to infection. Controls from challenge infection were used. **b** Hierarchical clustering of differentially abundant mRNAs performed on Euclidean distances using complete linkage. Cluster cut-offs (dendrogram resolution) were set to identify gene-sets with profiles interpretable in relation to the parasite lifecycle and between mice of different immune competence. Clusters are represented with color on the left-hand side of rows and additional numbering is used to refer to clusters (right)

We here show that upon infection with *E. falciformis*, which induces weight loss (Additional file 4: Figure S4) and intestinal pathology in mice, the host transcriptome undergoes drastic changes affecting more than 3000 individual mRNA profiles significantly (edgeR; glm likelihood-ratio tests corrected for multiple testing, false discovery rate [FDR] < 0.01). Statistical testing for differential abundance between infected and uninfected mice revealed that differences in mRNA abundance were more pronounced (both in magnitude and number of genes affected) at the two later time-points post infection (Table 2, Fig. 2a, Additional file 5: Figure S5). 325 mRNAs were differently abundant (FDR < 0.01) between controls and 3 dpi, 1804 mRNAs between controls and 5 dpi, and 2711 mRNAs between controls and 7 dpi. This leads to a combined set of 3453 transcripts responding to infection. Differentially abundant mRNAs early in infection (3 and 5 dpi) were not a mere subset of genes differentially abundant later in infection (7 dpi; Fig. 2a), which would be the case if the same genes were regulated throughout infection. Instead, the transcriptional profile of the mouse changes more fundamentally with different genes varying in abundance late compared to early in infection. This is in line with expression of cytokines as major regulators of immune responses [26, 32] against *E. falciformis* and with extended regulation of the mouse transcriptome upon infection [12].

**Table 2:** Number of mouse and *E. falciformis* mRNAs significantly differentially abundant in different comparisons (Contrasts)

Contrast	Number of *E. falciformis* mRNAs with FDR < 0.01	Number of mouse mRNAs with FDR < 0.01
NMRI 7 dpi vs. uninfected control		2711
NMRI 5 dpi vs. uninfected control		1804
NMRI 3 dpi vs. NMRI 7 dpi	1399	1322
C57BL/6 5 dpi vs. uninfected control		919
NMRI 7 dpi naïve vs NMRI 7 dpi challenge	0	857
NMRI 5 dpi vs. NMRI 7 dpi	2084	732
*Rag1* ^−/−^ vs C57BL/6		362
NMRI 3 dpi vs ctrl		325
C57BL/6 5 dpi naïve vs C57BL/6 5 dpi challenge	0	175
*Rag1* ^−/−^ 5 dpi vs control		42
NMRI 3 dpi naïve vs NMRI 3 challenge	1	18
NMRI 3 dpi vs. NMRI 5 dpi	103	0
NMRI 5 dpi vs. oocysts	3691	
Sporozoites vs. oocysts	3532	
NMRI 3 dpi vs. oocysts	3303	
NMRI 7 dpi vs. oocysts	3202	
NMRI 7 dpi vs. sporozoites	2663	
NMRI 5 dpi vs. sporozoites	1726	
NMRI 3 dpi vs. sporozoites	1705	
NMRI control vs. C57BL/6 control	13	

Empty cells indicate that comparison is not applicable

To further analyze the distinct responses early and late in infection, we performed hierarchical clustering on transcript abundance patterns at different time-points post infection (Fig. 2b). Three main sample clusters formed (dendrogram indicating similarities between columns at top of Fig. 2b). Immune deficient *Rag1* ^*−/−*^ mice, including infected *Rag1* ^*−/−*^ samples, show an expression pattern most similar to uninfected samples. This similarity between infected and non-infected *Rag1* ^*−/−*^ samples confirms the immune deficiency phenotype; a failure to react to infection in these mice, and suggests a strong influence of the adaptive immune system on overall transcriptional responses. Surprisingly, these patterns indicate that innate immune responses and other B- and T-cell independent processes play detectable though relatively small roles (mouse gene cluster 4; Mm-cluster hereafter, Fig. 2b) in shaping the mouse transcriptome upon *E. falciformis* infection.

### Responses to parasite infection differ between immunocompromised and immune competent mice

The self-limiting nature of *E. falciformis* infection and host resistance to reinfection ([32] and Fig. 1a) makes it interesting to analyze transcriptomes of immune competent hosts in depth. On 3 and 5 dpi, mRNAs of two gene clusters have overall high abundance in samples of all immune competent infected animals (Mm-clusters 1 and 2). Other mRNAs (Mm-clusters 3 and 4) show lowered abundance in all those infected samples.

Gene Ontology (GO) terms enriched among the mRNAs which become more abundant only early in infection (Mm-clusters 1 and 2) are, e.g., “stem cell population maintenance”, “mRNA processing”, and “cell cycle G2/M transition”, indicating tissue remodeling in the epithelium. This is expected in an infection which damages epithelial tissue [20, 26, 33], but the early onset of these reactions is noteworthy. In addition, terms such as “regulation of response to food” are enriched (Additional file 6: Table S1). This is interesting since weight losses and malnutrition are generally common during parasitic infections [34, 35], also in *Eimeria* spp. infections [33, 36, 37], and weight loss was also seen in the present study (Additional file 4: Figure S4).

Genes whose mRNA levels decreased in abundance upon infection (Mm-clusters 3 and 4) indicate induction of IL-1 and IL-6, which are involved in inflammation, including T- and B-cell recruitment and maturation, and broad acute phase immune responses (Additional file 6: Table S1). IL-6 has also been shown to support tissue repair and inhibit apoptosis after epithelial wounding [38]. In addition, IL-6 is linked to Th17 responses [39] which are known to play an important role in responses to *E. falciformis* [12, 26]. Further terms indicate a regulation of transforming growth factor-β (TGFβ) which is important for wound healing in intestinal epithelium [40], epidermal growth factor (EGF) and tumor necrosis factor (TNF), which regulate proliferation of epithelial cells and inhibit apoptosis in epithelial cells [41, 42]. Inhibition of Notch signaling, which is also highlighted by GO terms, has been shown to alter the composition of cell-types in the epithelium towards Paneth and Goblet-like cells [43].

Although speculative, several of the GO terms (e.g. “calcineurin-NFAT signaling cascade”, “Inositol-phosphate mediated signaling”, “Notch receptor processing” in addition to those mentioned above) annotated to genes whose mRNA levels change in abundance upon early infection (Mm-cluster 3 and 4) can be linked to explain fundamental mechanisms. Inositol signaling can lead to release of calcium and calcineurin-dependent translocation of NFAT to the nucleus; and there to activation of NFAT target genes in T-cells, but also many other cell types [44]. In addition, changes in the host epithelium do take place when cells are invaded by, e.g., *E. falciformis*, but also generally by pathogens, and this is reflected in the stem-cell and cell cycle-related GO terms described above for Mm-clusters 1 and 2. Further investigation of the role of the processes and molecules highlighted here will contribute to better understanding of epithelial responses to intestinal intracellular parasitic infection. Interestingly, in T- and B- cell deficient hosts, the same four groups of genes described above (Mm-clusters 1–4, Fig. 2b), which are responsible for these dominating responses in immune competent hosts show no differences between infected and non-infected immune deficient animals.

### Adaptive immune responses characterize late infection

Pronounced transcriptional changes in the mouse host occur late in infection in immune competent animals (Table 2 and Mm-cluster 5 in Fig. 2b). Annotated processes and functions (GO terms) for genes with increased abundance at 7 dpi reflect the expected onset of an adaptive immune response (Additional file 6: Table S1). As late as 5 dpi, genes responsible for these enrichments are still low on mRNA abundance. This confirms a strong induction of immune responses, particularly adaptive immune responses, between 5 and 7 dpi. This result is well in line with previously described immune responses to infection with *Eimeria* spp. [19–25].

### Protective responses occur earlier in challenge infected than in naïve hosts

Transcriptomes from three samples from early and late challenge infection show the same distinct profile of elevated mRNA abundance at 3, 5 and 7 dpi (Mm-cluster 6, Fig. 2b). The underlying mRNAs are highly enriched for GO terms for RNA processing, e.g., splicing, which indicated post-transcriptional regulation. In addition, terms for histone and chromatin modification are enriched (Additional file 6: Table S1). This, along with less oocyst shedding during challenge infection, suggests that protective immune responses in challenge infected animals are regulated both at the transcriptional and post-transcriptional level. The high abundance of these mRNAs at different time-points post infection in wild-type hosts (NMRI) further indicates that protective immunity is similar at these time-points. Possibly, induction and chronologic differences in challenge infected animals occur before 3 dpi. The completely cleared infection in some samples (Table 1; and unexpected clustering of e.g. NMRI_2ndInf_7dpi_rep2), apart from clearly demonstrating protection, also supports an early timing of this response upon challenge infection. However, the distinct shared profile at the investigated time-points (days 3, 5, and 7) does show that the protective response is still detectable at the transcriptional level several days after the challenge.

### A framework to interpret *E. falciformis* transcriptomes is provided by orthologues in the Coccidia *E. tenella* and *T. gondii*

To establish *E. falciformis* as a model for coccidian parasites, transcriptome profiles of orthologue genes from closely related parasites can help to draw parallels between lifecycle stages. This can be informative in predicting gene function and in analyzing evolutionary forces acting on the different lifecycle stages. Therefore, we performed correlation analysis between our *E. falciformis* transcriptome and RNA-seq transcriptomes from closely related parasites at corresponding stages of their lifecycles. Two datasets for the economically important chicken parasite *E. tenella* [45, 46] and one dataset of the model apicomplexan parasite *T. gondii* [47] were included. The latter was used because it is to date the only available dataset for the lifecycle of *T. gondii* for multiple stages within and outside of both an intermediate host and the definitive (cat) host, and it is therefore comparable with our data.

**Fig. 3:**
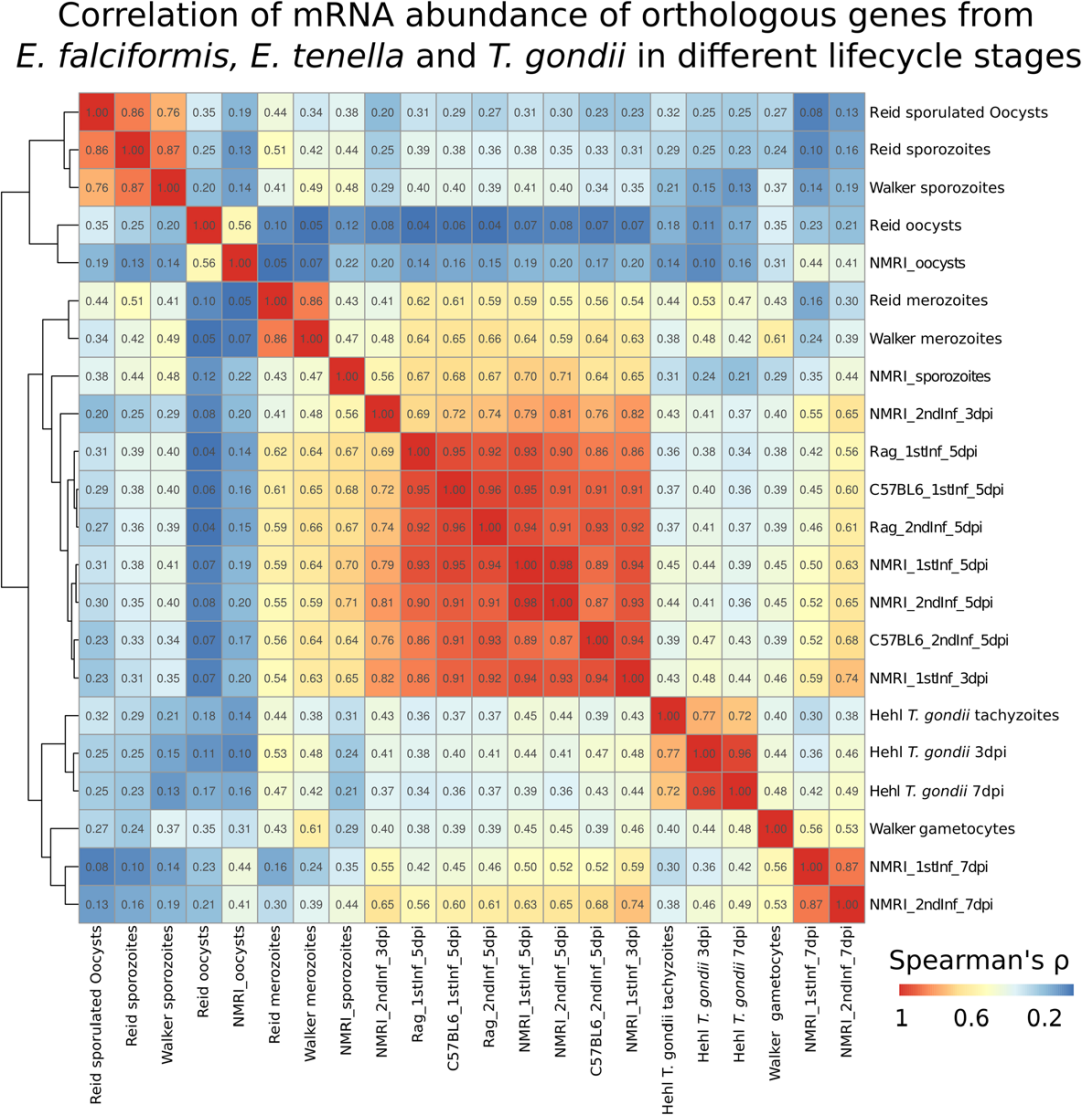
Correlations of *E. falciformis* mRNA abundance with ortholgues from other Coccidia. *E.falciformis* mRNA abundance was compared to that of orthologous genes of *E. tenella* [45, 46] and *T. gondii* [47]. Correlation coefficients (Spearman’s ρ) were clustered using complete linkage. *T. gondii* and *Eimeria* spp. “late infection” samples cluster together. *E. falciformis* early infection samples cluster with *E. tenella* merozoites. *E. falciformis* sporozoites cluster with *E. falciformis* early infection, whereas unsporulated oocysts cluster with *E. tenella* unsporulated oocysts

For all samples from these studies and our data, abundances of orthologous genes were correlated and Spearman’s coefficient was compared (Fig. 3). With the exception of sporozoites (see below), transcriptomes tend to be more strongly correlated (similar) between corresponding lifecycle stages of different parasite species than between stages in the same parasite species.

Orthologues in *E. tenella* and *E. falciformis* gamete stages (purified gametocytes and 7 dpi intestinal samples, respectively) are highly correlated in their expression across the two species, indicating conserved gene sets orchestrating sexual replication of the two parasites. Similarly, transcriptomes of *E. tenella* merozoites from both independent studies of that parasite are most similar to early *E. falciformis* samples, indicating similarity also during asexual reproduction. *E. falciformis* unsporulated oocyst transcriptomes share the highest similarity with those of unsporulated *E. tenella* oocysts.

*E. falciformis s*porozoites transcriptome profiles are more similar to *E. falciformis* early infection samples than to sporozoite transcriptomes of *E. tenella* orthologues*.* Similarities between sporozoites and early infection stages could be explained by similar biological processes, especially host cell invasion (and reinvasion by merozoites), being prepared or performed. Sporozoites are the only lifecycle stages in which orthologue mRNA abundance patterns show such dissimilarities to *E. tenella* and this might indicate a higher species specificity of the genes and processes in this invasive stage. This could be a result of virulence factors being expressed in this stage, which are known to undergo rapid gene family expansion, as seen in surface antigens (SAGs) in *E. falciformis* [10], *T. gondii* [48], *Neospora caninum* [49], and other *Eimeria* spp. [45], or *var.* genes in *Plasmodium falciparum* [50].

Below we provide a detailed description of the *E. falciformis* transcriptome, including a discussion of genes which have been shown to be important in closely related parasites such as *E. tenella* and *T. gondii.*

### *Overall transcriptional changes in the lifecycle of* E. Falciformis

**Fig. 4:**
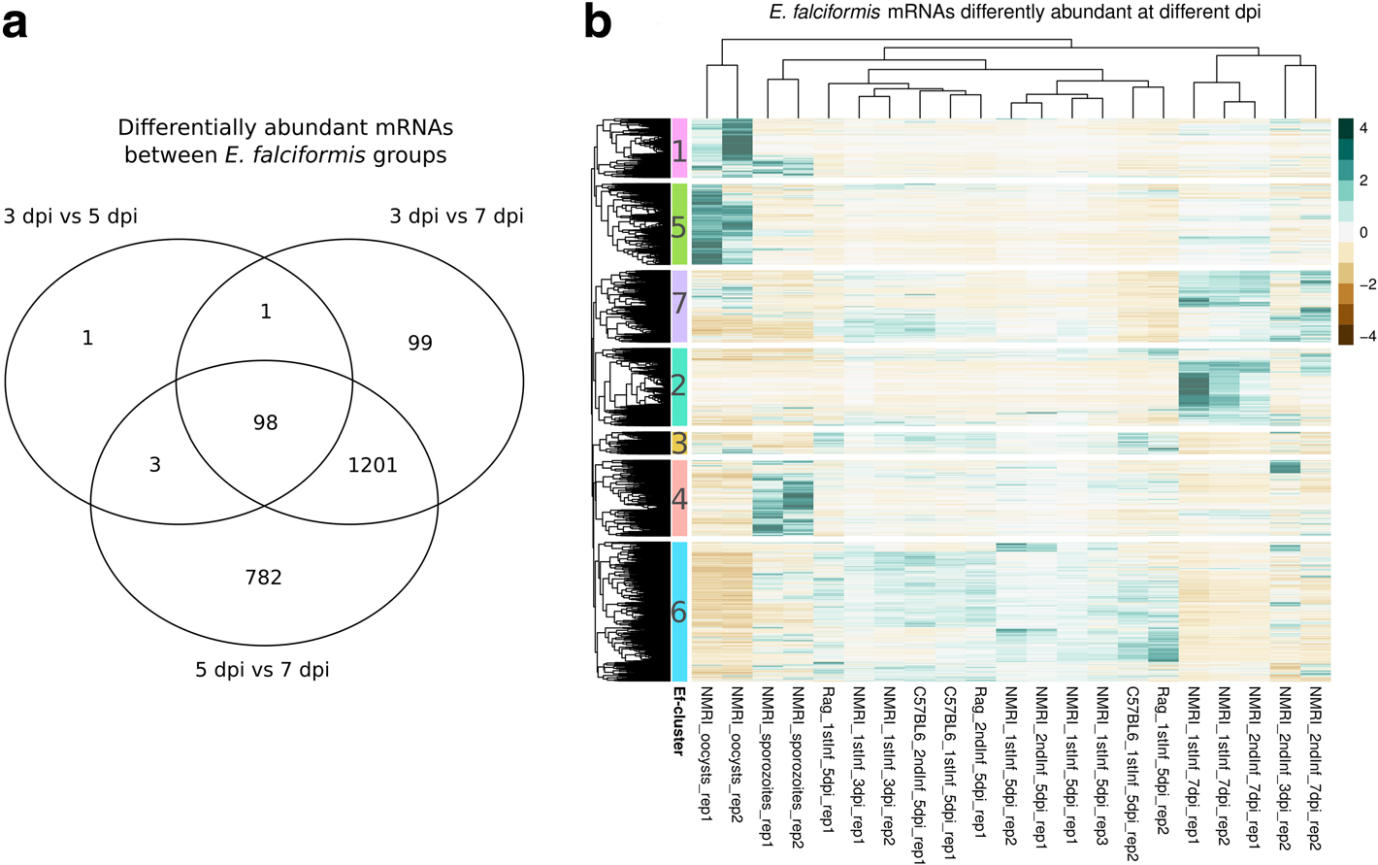
Differentially abundant *E. falciformis* mRNAs and clustering thereof. **a** Venn diagram visualizes the overlap between genes showing differential abundance (FDR < 0.01; edgeR glm likelihood-ratio tests) between intracellular stages at 3 dpi, 5 dpi and 7 dpi. **b** Hierarchical clustering of abundance profiles for differentially abundant mRNAs performed on Euclidean distances using complete linkage. Cluster cut-offs (dendrogram resolution) were set to identify gene-sets with profiles interpretable in relation to the parasite lifecycle. Clusters are represented with color on the left-hand side of rows and additional numbering is used to refer to clusters (right)

Similar to the host transcriptome, differences in parasite mRNA abundance were mostly observed between late and early infection. Between 3 and 5 dpi 103 mRNAs were differently abundant (edgeR likelihood ratio tests on glms; FDR < 0.01), whereas between 3 and 7 dpi 1399 mRNAs, and between 5 and 7 dpi 2084 mRNAs were differentially abundant (Fig. 4a, Table 2, Additional file 5: Figure S5). We therefore define transcriptomes as distinct at a threshold of >1000 parasite genes being differently expressed given the statistical power of our experiment (and i.e. regard the ~100 genes in 3 dpi versus 5 dpi less relevant for our analysis). Hierarchical clustering resulted in seven different gene clusters, with differently pronounced profiles in different lifecycle stages (sample clusters). Confirming the analysis based on significant thresholds (differential abundance), clustering did not separate samples from 3 and 5 dpi and we thus refer to these as “early infection” and 7 dpi as “late infection”. Distinct abundance differences (>1000 genes differentially expressed and separated by sample clustering) define early infection with a single cluster of genes (parasite gene cluster 6, “Ef-cluster 6” hereafter, Fig. 4b). At those time-points asexual reproduction takes place [11, 13]. Two separate gene clusters define late infection (7 dpi, Ef-clusters 2 and 7). The separation of these genes into two gene clusters was driven by slightly different expression profiles during other lifecycle stages while being mainly characterized by very strong expression at 7 dpi. In these samples we assume gametocytes to be present due to the peak of oocyst shedding 1 day later (Fig. 1a) [11] and similarity of these transcriptomes with purified *E. tenella* gametocytes (Fig. 3). The extracellular stages, sporozoites (Ef-cluster 4) and unsporulated oocysts (Ef-clusters 1 and 5) are clearly distinguished by high mRNA abundance. In order to assess the biological relevance of these patterns, we applied enrichment analyses for GO terms and “gene family conservation profiles” based on earlier annotations [10].

### *Sporozoites express genes which are evolutionarily unique to* E. Falciformis

Sporozoites are in our study released from oocysts in vitro, after which they are capable of invading host cells. We suggest that the requirement for proteins which mediate motility and other invasion processes are reflected by their mRNA levels in the transcriptome. Due to the “host-mRNA free” nature of transcriptomes generated from sporozoites raised in vitro and deep sequencing it was possible to assess those transcripts even at relatively low mRNA expression levels observed for some of them [51] (Additional file 5: Figure S5).

We find that *E. falciformis* sporozoites are defined by a group of genes (Ef-cluster 4, Fig. 4b) that is largely specific to *E. falciformis* (Table 3). This indicates that *E. falciformis* does not share with other species many of the abundant sporozoite genes so far described for those Coccidia. Interestingly, five out of 12 SAG gene transcripts predicted for *E. falciformis* [10] are typical for sporozoites. SAG proteins, divergent or unrelated between species, are thought to be involved in host cell attachment and invasion, and possibly in induction of immune responses in other apicomplexan species [45, 49, 52–56]. In total, mRNAs encoding ten SAGs were detected as differentially abundant in our data, but in other lifecycle stages than sporozoites. Such expression of particular SAGs in stages other than sporozoites has been reported for *E. tenella* [57]*.* Genes also receiving attention as potential virulence factors in *E. tenella* are rhoptry kinases (RopKs) [58]. Transcripts of two out of ten *E. falciformis* orthologues of RopKs are highly abundant in sporozoites (Ef_cluster 4). Also in *E. tenella* some RopKs are expressed predominantly in sporozoites and have been shown to be differentially expressed compared to *E. tenella* intracellular merozoite stages [59]. For genes with orthologues known to be important in other Coccidia, e.g., SAGs and RopKs, orthologues indicate a molecular function, but the biological relevance of their expression in *E. falciformis* remains unclear.

**Table 3:** Enrichments and underrepresentation of species or species-group orthologues in *E. falciformis* gene clusters (from Fig. 3b)

*E. falciformis* cluster	Conservation category	Odds ratio	*p*-value	FDR
Ef-cluster 2 (up at 7 dpi)	Conserved	0.67	9.03E-06	1.90E-04
Ef-cluster 4 (up in sporozoites)	Conserved	0.72	2.44E-04	1.71E-03
Ef-cluster 7 (up at 7 dpi)	Conserved	1.72	1.11E-10	4.65E-09
Ef-cluster 2 (up at 7 dpi)	ApicomplexaC	0.45	1.84E-04	1.71E-03
Ef-cluster 5 (up in oocysts)	ApicomplexaC	1.86	3.76E-05	5.26E-04
Ef-cluster 4 (up in sporozoites)	*E. falciformis*	3.05	2.38E-04	1.71E-03
Ef-cluster 1 (up in oocysts)	*Eimeria*	0.68	1.83E-03	9.59E-03
Ef-cluster 6 (up in early inf)	Apicomplexa	1.46	1.11E-03	6.64E-03

Odds ratios higher than one indicate enrichment and smaller than one indicate underrepresentation. Conservation categories were chosen as previously described [10]. Only significant results (FDR < 0.05) are shown

For the overall biological functions of sporozoite genes (Ef-cluster 4), GO enrichment data suggests ATP production and biosynthesis processes as dominant features (Additional file 7: Table S2). In addition, this invasive stage is characterized by "maintenance of protein location in cell" and GO terms which indicate similar biological functions. Possibly, this reflects control of microneme or rhoptry protein localization as sporozoites prepare for invasion. Sporozoites therefore display a transcriptome indicative of large requirements for ATP and production of known virulence factors such as SAG and RopKs and are characterized by expression of species specific genes.

Genes typical for the sporozoite stage displayed a species specific profile with the respective gene families absent outside *E. falciformis* (Table 3). This mirrors our analysis of orthologous genes, in which sporozoites were the only lifecycle stage not displaying strong cross-species correlation in their transcriptome. This suggests that traits involved in host cell invasion may have evolved quickly and rapidly became specific for a parasite in its respective host species or target organ niche.

### Growth processes dominate the transcriptome during asexual reproduction

Invasion of epithelial cells by sporozoites is followed by asexual reproduction leading to a massive increase in parasite numbers between 3 and 5 dpi, when several rounds of schizogony take place in a somewhat unsynchronized fashion [11, 13]. In early infection, and similar to sporozoites, mRNAs annotated for biosynthetic activity are enriched, but different genes/mRNAs are contributing to enrichment of similar GO terms compared to sporozoites (Additional file 7: Table S2). Enrichment of terms referring to replication and growth-related processes (biosynthesis) highlights the parasite’s expansion during schizogony.

Amongst early infection high abundance mRNAs, we found four out of ten RopKs which are predicted in *E. falciformis* [10]. This is the largest number of RopKs in any one group of differentially abundant mRNAs in our analysis and they constitute a statistically significant enrichment (Fisher’s exact test; *p* < 0.001). Three of these have orthologues in *T. gondii*: ROP41, ROP35 and ROP21 [60–63]. Our data gives a first overview of expression patterns for *E. falciformis* RopKs and offer a good starting point for functional analysis of these virulence factors in *Eimeria* spp.

### Gametocyte motility dominates the transcriptome late in infection

Two *E. falciformis* gene clusters show a distinct profile characterized by high mRNA abundance on 7 dpi (Ef-clusters 2 and 7; Fig. 4b). Both clusters display low mRNA abundance in other lifecycle stages, especially in oocysts and sporozoites. Enriched GO terms such as "movement of cell or subcellular component" and “microtubule-based movement” along with terms suggesting ATP production (e.g. “ATP generation from ADP”) indicate the presence of motile and energy demanding gametocytes in these samples. Peptide and nitrogen compound biosynthetic processes along with “chitin metabolic process” (Additional file 7: Table S2) also suggest that the parasite produces building blocks for oocysts and their walls in this stage. Our data confirms findings of Walker et al. (2015) in *E. tenella* gametocytes: these authors also identified cytoskeleton related and transport processes as upregulated in gametocytes compared to merozoites or sporozoites [46].

### Oocysts are characterized by cell differentiation and DNA replication processes

Oocysts are the infective stage in the lifecycle of Coccidia. They are shed with feces as unsporulated, “immature”, capsules and in the environment they undergo sporulation – meiotic and mitotic divisions [8] – and become infective. Our oocysts were purified in the unsporulated stage from passage in lab mice. Two expression clusters of mRNA are highly abundant in this stage (Ef-clusters 1 and 5; Fig. 4b). One of these oocyst gene sets (Ef-cluster 5) is enriched for apicomplexan-shared orthologues (Table 3) and for GO terms such as “DNA repair”, “protein modification process” and “cell differentiation”, supporting that expected sporulation processes have been initiated. The same cluster is also the only cluster which is enriched for transmembrane domains (Fisher’s exact test, FDR < 0.001).

### *E. falciformis* does not respond plastically to differences in the host transcriptome

We show that infections of *E. falciformis* in its natural host, the house mouse, display a chronological pattern independent of the immune status of the host. This suggests genetic canalization of the number and timing of asexual reproductive cycles during schizogony. Similar observations have been reported before for a closely related parasite strain [17]. Beyond developmental timing, parasites appear to lack strategies for most efficient host-interactions in response to the host’s immune status. This is supported by the lack of differences in parasite transcriptomes from immune competent and immune deficient hosts, or from naïve and challenge infected hosts (Fig. 4b).

In its core our finding of a lack of transcriptional plasticity is a negative result: we can – given our experimental design and statistical power – not reject our null hypothesis, which is the absence of differences. It is impossible to prove a negative [64]. However, using the changes across the parasite lifecycle as a benchmark we can state that any change in the parasite transcriptome would be so minute to be very unlikely to correspond to an altered “infection program” or strategy.

Only recently have transcriptomes been used to investigate plasticity in “infection programs”, which parasites induce as a response to host signals. Since gene expression is orchestrated by the genetic makeup of an organism, plasticity in transcription – when it occurs – is likely to be an adaptation which allows the parasite to react on host stimuli and to produce an altered phenotype. We here suggest that it is useful to distinguish between such plastic (responsive) transcription programs and more “passive” forms of phenotypic change imposed on the parasite without being controlled at the transcriptional level. In our case, the extent of oocyst shedding – probably an important component of parasite fitness – appears to be attributable to “unbuffered” host impact. In a Nematode, the presence of phenotypic plasticity has for example been shown to lack a transcriptional basis [65], and could therefore be regarded “passive” or “unbuffered”. In contrast, unicellular *Entamoeba* spp. infections of variable pathogenicity (i.e. phenotypic plasticity) did indeed manifest in transcriptional differences between the parasites under various in vitro conditions [66]. Among apicomplexan parasites, different infection programs with distinct transcriptional profiles have been proposed: in *Plasmodium* spp., the parasite’s transcriptome is distinct in different mouse genotypes (BALB/c and C57BL/6) and tissues within one genotype [67], hence demonstrating the capability for – likely adaptive – plasticity in this parasite. Similarly and even more closely related to *Eimeria* spp., the coccidian *T. gondii* forms dormant tissue cysts (bradyzoites), a process induced by and depending on the host environment [68], and involving large changes in parasite transcriptomes [69]. In addition, *T. gondii* is capable of infecting all studied warm-blooded vertebrates and all nucleated cells in those animals [70] suggesting parasite plasticity in different host environments also in the tachyzoite stage.

A switch from epithelial remodeling and innate immune processes to adaptive immune responses in the immune competent host, between 5 and 7 dpi, is paralleled by a switch from asexual to sexual reproduction of *E. falciformis* irrespective of host immune status. This contemporaneity might be an evolutionary adaptation of the parasite to host responses in order to complete its lifecycle before the host environment becomes hostile. Such a response could be based on genetically canalized developmental timing or the parasite sensing an immune challenge and establishing a reaction plastically. Our results on parasite development support a genetically canalized developmental timing. Beyond this developmental timing, the severity of *E. falciformis* infection (measured as the extent of oocyst shedding) varies between hosts of different immune competence.

We propose that adaptive plasticity would be identified as a transcriptional response. Since the parasite’s transcriptome in an immune deficient host cannot be distinguished from the one in an immune competent host, we suggest that *E. falciformis* follows a non-plastic, and instead genetically canalized program in the mouse host. We therefore conclude that *E. falciformis* cannot plastically adjust infection strategies to optimize exploitation of hosts which vary in susceptibility.

## Conclusion

In this dual transcriptome study, we provide a thorough description of transcriptional responses in mice to infection with *E. falciformis,* and corresponding parasite transcriptomes. The mouse epithelial transcriptome of naïve, immune competent mice changes upon infection. Responses in wild-type challenge infected hosts suggest strong regulation both at the transcriptional level and in RNA processing. In contrast, these patterns are missing in immunocompromised animals which instead show a minimal transcriptional response to infection, demonstrating the host dependence of mature T- and B-cells for a natural response to this coccidian parasite.

For the first time we also describe transcriptomes of *E. falciformis* for multiple stages of the lifecycle within and outside of the host. Parasite transcriptomes are not distinguishable between hosts of different immune competence, demonstrating lack of plasticity at the gene expression level. Two independent assessments of evolutionary conservation show that invasive sporozoites possess the most species-specific transcriptomes in the *E. falciformis* lifecycle. We therefore suggest that excysted sporozoites express most of the genes involved in host-parasite co-evolutionary processes, which accelerate divergence and may determine niche specificity.

Taken together, we propose that *E. falciformis* follows a genetically predetermined path rather than responding to cues from the host, such as differences in immune responses. We further suggest that analyzing plasticity in parasites and comparing this between different host genotypes or species can be a useful tool to understand co-evolution of parasites and hosts: plastic responses can potentially be linked to the evolution of a generalist parasitic life-style infecting multiple different hosts or tissues, the lack or loss of plasticity, in contrast, to niche or host specitivity. We emphasize that gene expression is not necessarily a product of plastic host-parasite interactions, especially not in the parasite, but may instead follow genetically determined programs. The question whether relationships between genotype and phenotype are generally less plastic in unicellular compared to multicellular parasites requires further research.

## Methods

### Mice, infection procedure and infection analysis

Three strains of mice were used in our experiments: NMRI, C57BL/6 (Charles River Laboratories, Sulzfeld, Germany), and *Rag1* ^*−/−*^ on C57BL/6 background (obtained from German Rheumatism Research Centre, Berlin). *Rag1* ^*−/−*^ −mice are deficient in T- and B-cell maturation. Animals where infected as described by Schmid et al. [71], but tap-water was used instead of PBS for administration of oocysts. Briefly, NMRI mice were infected two times, which will be referred to as naïve and challenge infection. For the naïve infection, 150 sporulated oocysts were administered in 100 μL water by oral gavage. During the naïve infection of 52 mice, all animals were weighed every day. On day zero, before infection, as well as on 3 dpi, 5 dpi and 7 dpi, ceca from 3 to 4 sacrificed mice per time-point were collected. Epithelial cells were isolated as described in [71], using a protocol which generated epithelial cells with 90% purity. For challenge infection, mice recovered spontaneously and were after 4 weeks challenge infected. Recovery was monitored by weighing and visual inspection of fur. For the challenge infection, 1500 sporulated oocysts were applied by oral gavage in 100 μL water (a higher dose was necessary to establish a challenge infection). Tissue from three to four mice per replicate was pooled for both non-reinfection control (referred to as day 0 of challenge infection) and for all other samples. *Rag1* ^*−/−*^ mice and the background C57BL/6 strain control mice were also subjected to naïve and challenge infections with 10 sporulated oocysts in 100 μL water in both cases. Samples were taken on day 0 (pre-infection control) and 5 dpi in both naïve and challenge infections of these mice and were otherwise treated as described above for NMRI mice. Oocyst shedding was determined from eight NMRI mice in naïve infection and four challenge infected, from 15 naïve Rag1^−/−^ and C57BL/6 mice respectively, and from nine challenge infected Rag1^−/−^ and C57BL/6 mice, respectively. Overall oocyst output was compared using Mann-Whitney U-test in R [72].

### Oocyst purification for infection, sequencing and quantification

Oocysts for infection were purified by NaOCl flotation of mouse feces stored in potassium dichromate, in which oocysts for infection were allowed to sporulate at room temperature for at least 5 days. During the patency phase, feces of mice were collected and oocysts were flotated using saturated NaCl-solution. The oocyst output was quantified using the McMaster chamber. For sequencing, unsporulated oocysts were purified twice per day from feces of NMRI mice on 8–10 dpi, and immediately subjected to RNA purification. The strain “*E. falciformis* Bayer Haberkorn 1970” was used for all infections and parasite samples, it is maintained through passage in NMRI mice in our facilities as described previously [71].

### Sporozoite isolation

Sporocysts were isolated according to the method of [73] with slight modifications. Briefly, not more than 5 million sporulated oocysts were resuspended in 0.4% pepsin solution (Applichem), pH 3, and incubated at 37 °C for 1 h. Subsequently, sporocysts were isolated by mechanical shearing using glass beads (diameter 0.5 mm) and a vortex mixer, washed and separated from oocyst cell wall components by centrifugation at 1800 g for 10 min. Sporozoites were isolated from sporocysts by in vitro excystation. For this, sporocysts were incubated at 37 °C in DMEM containing 0.04% tauroglycocholate (MP Biomedicals) and 0.25% trypsin (Applichem) for 30 min. Released sporozoites were purified in cellulose columns as described in [74].

### RNA extraction and quantification

For RNA-seq, total RNA was isolated either from infected epithelial cells, sporozoites, or unsporulated oocysts using Trizol according to the manufacturer’s protocol (Invitrogen). In addition, unsporulated oocysts in Trizol were treated by mechanical shearing using glass beads for at least 20 min under frequent microscopic inspection. Purified RNA was used to produce an mRNA library using Illumina’s TruSeq RNA Sample Preparation guide. This kit uses poly-T priming and we thus do not assess non-polyadenylated transcripts like those derived from the apicoplast genome. For qPCR, uninfected and infected epithelial cells from 3, 5 and 7 dpi were isolated as described above and cells were stored in 1 mL Trizol at −80 °C. Total RNA was isolated using the PureLink RNA Mini Kit (Invitrogen) and immediately reverse transcribed into cDNA using the Superscript III Platinum Two Step qRT-PCR Kit (Thermo Fisher Scientfic). These RNA preparations were used for RT-qPCR of *Eimeria* 18S and creation of a mouse gene reference index. For the reference index, the mouse genes cytochrome c-1 (Cyc), peptidylprolyl isomerase A (Ppia) and peptidylprolyl isomerase B (Ppib) were amplified using the primers Cyc1_qPCR_f (5′- CAGCTACCATGTCACAAGTAGC-3′) and Cyc1_qPCR_r (5′-ACCACTTATGCCGCTTCATG -3′); Ppib_qPCR_f (CAAAGACACCAATGGCTCAC) and Ppib_ qPCR_r (5′-TGACATCCTTCAGTGGCTTG-3′); Ppia_qPCR_f (5′-ACCGTGTTCTTCGACATCAC-3′) and Ppia_qPCR_r (5′-ATGGCGTGTAAAGTCACCAC-3′), respectively. The *E. falciformis* 18S gene was amplified using the primers Ef18s_for (5′-ACAATTGGAGGGCAAGTCTG-3′) and Ef18s_rev (5′-AAACACCAACAGACGCAGTG-3′).

After initialization at 50 °C for 2 min followed by activation of enzymes at 95 °C, 40 amplification cycles consisting of denaturation at 95 °C for 15 s and combined annealing and elongation at 60 °C for 60s were performed. After each cycle the fluorescent signal was measured. A reference index was constructed taking the cube route of the multiplied crossing threshold (ct)-values for the three mouse genes. This composite “index ct-value” was used to calculate the ct difference (delta-ct) of the *E. falciformis* 18S gene. The lowest of these values was set as reference for calculation of how much more *E. falciformis* 18S RNA was detected compared to the level of background noise in the sample with the lowest value leading to delta-delta ct, or “noise normalized” ct-values. The number of transcripts above noise level was calculated taking these values as exponents to the base two. The procedure was performed in triplicate for each experimental group. A linear model was constructed in R [72] to predict these noise normalized delta-ct values by day post infection (dpi) and type of infection (naïve or challenge infected). This model excludes measurements at 0 dpi infection as background noise.

### Sequencing and quality assessment

cDNA libraries were sequenced on either GAIIX (13 samples) or Illumina Hiseq 2000 (14 samples) platforms after preparation in a total of four experimental batches as specified in Table 1. A fastq_quality_filter (FASTQ-toolkit, version 0.0.14, available at https://github.com/agordon/fastx_toolkit) was applied to Illumina Hiseq 2000 samples using a phred score of 10. We intentionally did not use a stringent trimming before mapping to genome assemblies as the mapping process itself has been shown to be a superior quality control [75].

### Alignment and reference genomes

The *Mus musculus* mm10 assembly (Genome Reference Consortium Mouse Build 38, GCA_000001635.2) was used as reference genome for mapping and corresponding annotations were used for downstream analyses. The *E. falciformis* genome [10] was downloaded from ToxoDB [48]. For mapping, mouse and parasite genome files were merged into a combined reference genome, and files including mRNA sequences from both species were aligned against this reference using TopHat2, version 2.0.14, [76] with the option –G specified, and Bowtie2, version 1.1.2, [77]. This was done to avoid spurious mapping in ultra-conserved genomic regions. Single-end and pair-end sequence samples were aligned separately with library type ‘fr-unstranded’ specified for pair-end samples. Bam files were used as input for the function “featureCounts” from of the R package “Rsubread” [78]. All subsequent analyses were performed in R [72].

### Differential mRNA abundance, data normalization and sample exclusions

After import of data to R, mouse and parasite data was separated using transcript IDs and analyzed, including normalization, separately. For each species, count data was normalized using the R-package edgeR version 3.16.2 [79] with the upperquartile normalization method. This raw data underlying our study is available as supplementary data S1. Briefly, genes with below an overall of 3000 reads (mouse) and 100 reads (*E. falciformis*) summed over all samples (libraries) were removed and normalization factors were calculated for the 75% quantile for each library. This normalization is suitable for densities of mapping read counts which follow a negative binomial distribution. We excluded samples NMRI_2nd_3dpi _rep1 and NMRI_2nd_5dpi_rep2 due to low parasite contribution (0.012% and 0.023%) to the overall transcriptome. Technically, this exclusion made it possible to obtain parasite read counts in agreement with a negative binomial distribution. Both excluded samples are from challenge infection and it is likely that the infected mice were immune to re-infection. One additional sample (NMRI_1stInf_0dpi_rep1) was excluded because the uninfected control showed unexpected mapping of reads to the *E. falciformis* genome (0.033%). As samples and individual replicates were sequenced in batches to different depth and using different instrumentation (Table 1) we performed multidimensional scaling of samples as quality controls using “plotMDS”. We also plotted mean expression vs. difference (MA) plots using “plotSmear”. Both functions are provided in the R package edgeR v 3.16.2 [79].

### Testing of differentially abundant mRNAs and hierarchical clustering

We also used edgeR v 3.16.2 [79] further to fit generalized linear models (GLMs with a negative binomial link function) for each gene (glmFit) and to perform likelihood ratio tests for models with or without a focal factor (glmLRT) using the “alternate design matrix” approach specifying focal contrasts individually. Tested contrasts comprised for the mouse a) infections at each time-point versus uninfected controls, b) corresponding time-points between different mouse strains and c) corresponding time-points and mouse strains for naïve and challenge infection. Since the control sample for infection in naïve NMRI mice was removed from the analysis (see above), the two uninfected replicates from challenge infection were used as uninfected controls in all NMRI mouse analyses. For the parasite, contrasts were set between a) all different stages of the lifecycle, as well as b) and c) as above (see also results in Table 2).

Mouse mRNAs which responded to infection or were differently abundant at different dpi (0 vs “any dpi” or “any dpi” vs “any dpi”; see Table 2) and *E. falciformis* genes showing differences between any lifecycle stage (oocysts versus sporozoites, or either of those versus “any dpi” or “any dpi” versus “any dpi”) were selected and used for hierarchical clustering. Hierarchical clustering was performed using the complete linkage method based on Euclidean distances between Z-scores (mRNA abundance values scaled for differences from mean over all samples of each gene in units of standard deviations).

### Enrichment tests and evolutionary conservation test

Gene Ontology (GO) enrichment analysis was performed using the R package topGO with the “weight01” algorithm and Fisher’s exact tests. We additionally performed a correction for multiple testing on the returned *p*-values (function “p.adjust” using the BH-method [80]). Similarly, a Fisher’s exact test and corrections for multiple testing were used to test for overrepresentation of transcripts with a signal sequence for entering the secretory pathway or containing transmembrane domains (as inferred using Signal P) which are predicted for the *E. falciformis* genome [10]. Evolutionary conservation of gene families was analyzed based on categories from [10] which are as follows: i) *E. falciformis* specific, ii) specific to the genus *Eimeria*, compiled by an analysis of *E. falciformis*, *E. maxima* and *E. tenella*, iii) Coccidia: *Eimeria* plus *T. gondii* and *Neospora caninum*, iv) Coccidia plus *Babesia microti*, *Theileria annulata*, *Plasmodium falciparum* and *Plasmodium vivax* v) the same apicomplexan parasites as in iv plus *Cryprosporidium hominis*, vi) universally conserved in the eukaryote super-kingdom inferred from an analysis of *Saccharomyces cerevisiae* and *Arabidopsis thaliana*. These categories were tested for overrepresentation in parasite gene clusters with particular patterns described in the text using Fisher’s exact-tests. Resulting p-values were corrected for multiple testing using the procedure of Benjamini and Hochberg [80] and reported as false discovery rates (FDR).

### Correlation analysis of apicomplexan transcriptomes

Transcriptome datasets from [45, 46] and [47] were downloaded from ToxoDB [48]. Orthologues between *E. falciformis*, *E. tenella* and *T. gondii* were compiled as in [10] and only 1:1:1 orthologue triplets were retained for analysis, as multi-paralog gene-families might contain members showing divergent evolution of gene-expression due to neo/sub functionalization. Mean mRNA abundances per lifecycle stage were used for samples from our study. Spearman’s correlation coefficients for expression over different samples in all studies and over different species represented by their orthologues were determined. Hierarchical clustering with complete linkage was used to cluster resulting correlations coefficients.

## Additional files

Additional file 1: Figure S1. Ordinations on mouse and parasite transcriptomes. The results of multidimensional scaling analyses are displayed for mouse and *E. falciformis* using different labels to allow comparisons. (PNG 741 kb)

Additional file 2: Figure S2. Controls for the properties of mRNA abundance distributions after setting different abundance thresholds per mRNA over all samples. (PNG 6538 kb)

Additional file 3: Figure S3. Mouse mRNA abundance in late *E. falciformis* infection versus uninfected controls, assessed by both RNA-seq (present data) and microarray. Mouse data from 7 dpi (RNA-seq) and 6 dpi. In both experiments, NMRI mice were infected with the same *E. falciformis* isolate. Even with one day difference in sampling, mouse transcriptomes show a strong correlation. The line depicted for visualization corresponds to generalized additive model using penalized regression splines. (PNG 1321 kb)

Additional file 4: Figure S4. Weight loss of mice during *E. falciformis* infection. Mouse weight is shown as a percentage relative to weight at the time of infection. Infection dose for NMRI was 150 oocysts in naïve infection and 1500 in challenge infection. For C57BL/6 and Rag1−/− dose was 10 oocysts in both naïve and challenge infection. Bars indicate standard error for three or four replicates. (JPEG 344 kb)

Additional file 5: Figure S5. Mean expression level vs. difference in between experimental groups. Mean expression levels are plotted in average counts per million (AverageCPM) vs. the log-Fold change for a particular comparison. Genes showing significant expression differences at a false discovery rate (FDR) threshold <0.01 are highlighted in red. Data for transcripts with zero abundance in one library is indicated using a “smearing” for respective AverageCPM values. (JPEG 2311 kb)

Additional file 6: Table S1. GO terms enriched in Mm-clusters in Fig. 2b. (CSV 50 kb)

Additional file 7: Table S2. GO terms enriched in Ef-clusters in Fig. 4b. (CSV 18 kb)
