# Supplementary figures and images for: Dual RNA-seq reveals no plastic transcriptional response of the coccidian parasite Eimeria falciformis to host immune defenses
Source: BMC Genomics. 2017 Sep 5;18:686. doi: 10.1186/s12864-017-4095-6 (PMC5584376; doi:10.1186/s12864-017-4095-6)

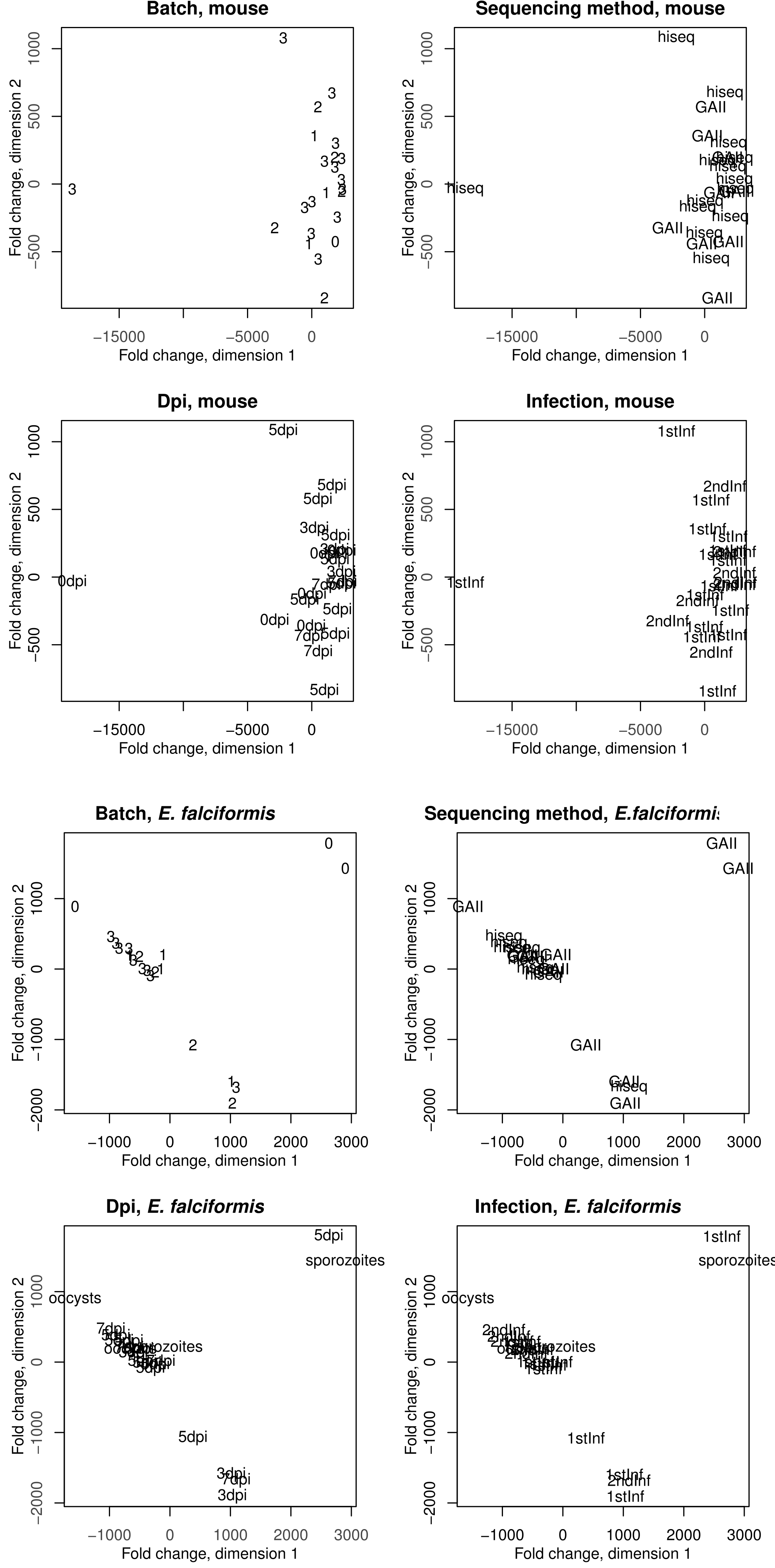

Supplement: Supplementary file 1 — Ordinations on mouse and parasite transcriptomes. The results of multidimensional scaling analyses are displayed for mouse and E. falciformis using different labels to allow comparisons. (PNG 741 kb) [file 12864_2017_4095_MOESM1_ESM.png]

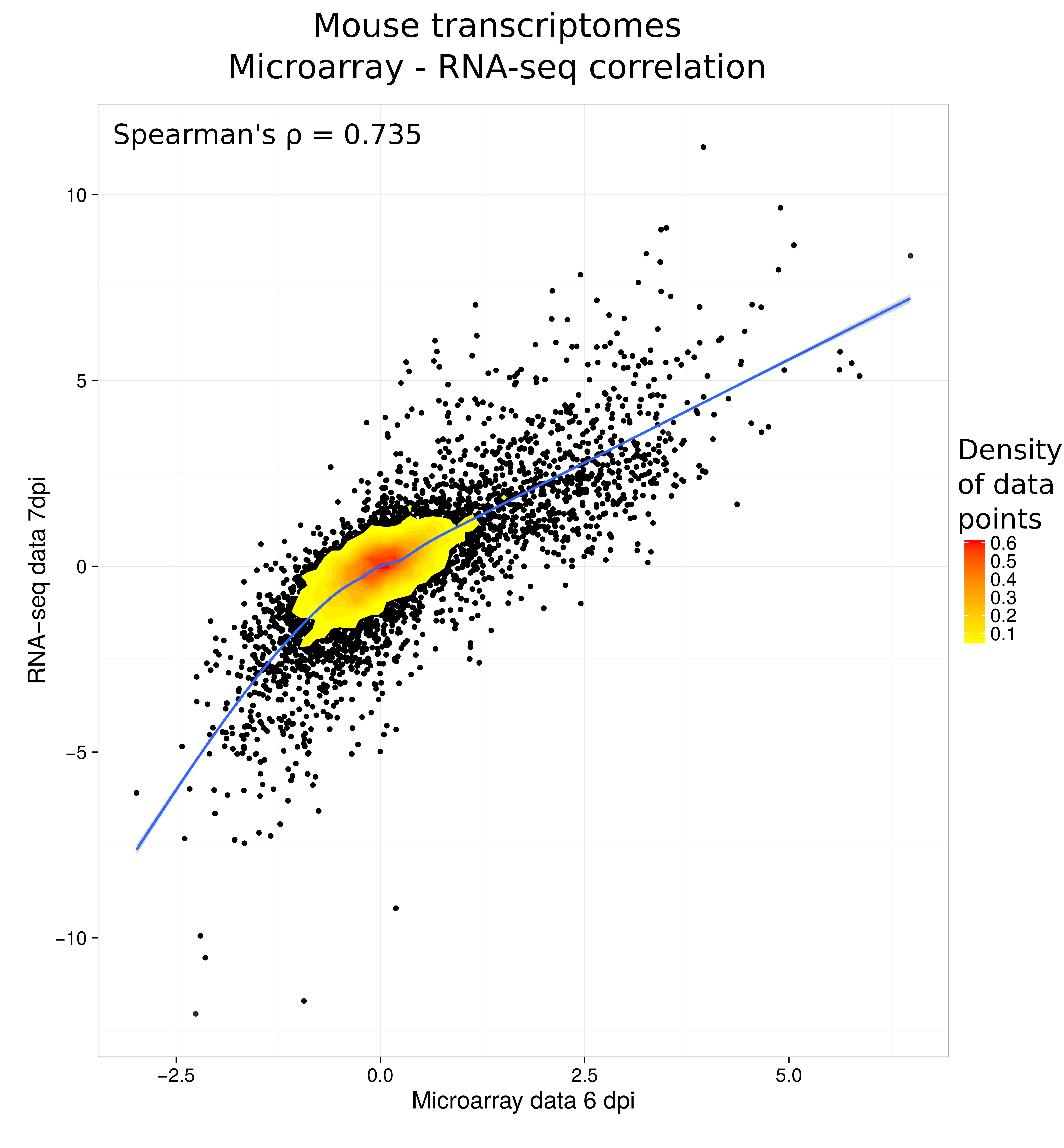

Supplement: Supplementary file 3 — Mouse mRNA abundance in late E. falciformis infection versus uninfected controls, assessed by both RNA-seq (present data) and microarray. Mouse data from 7 dpi (RNA-seq) and 6 dpi. In both experiments, NMRI mice were infected with the same E. falciformis isolate. Even with one day difference in sampling, mouse transcriptomes show a strong correlation. The line depicted for visualization corresponds to generalized additive model using penalized regression splines. (PNG 1321 kb) [file 12864_2017_4095_MOESM3_ESM.png]

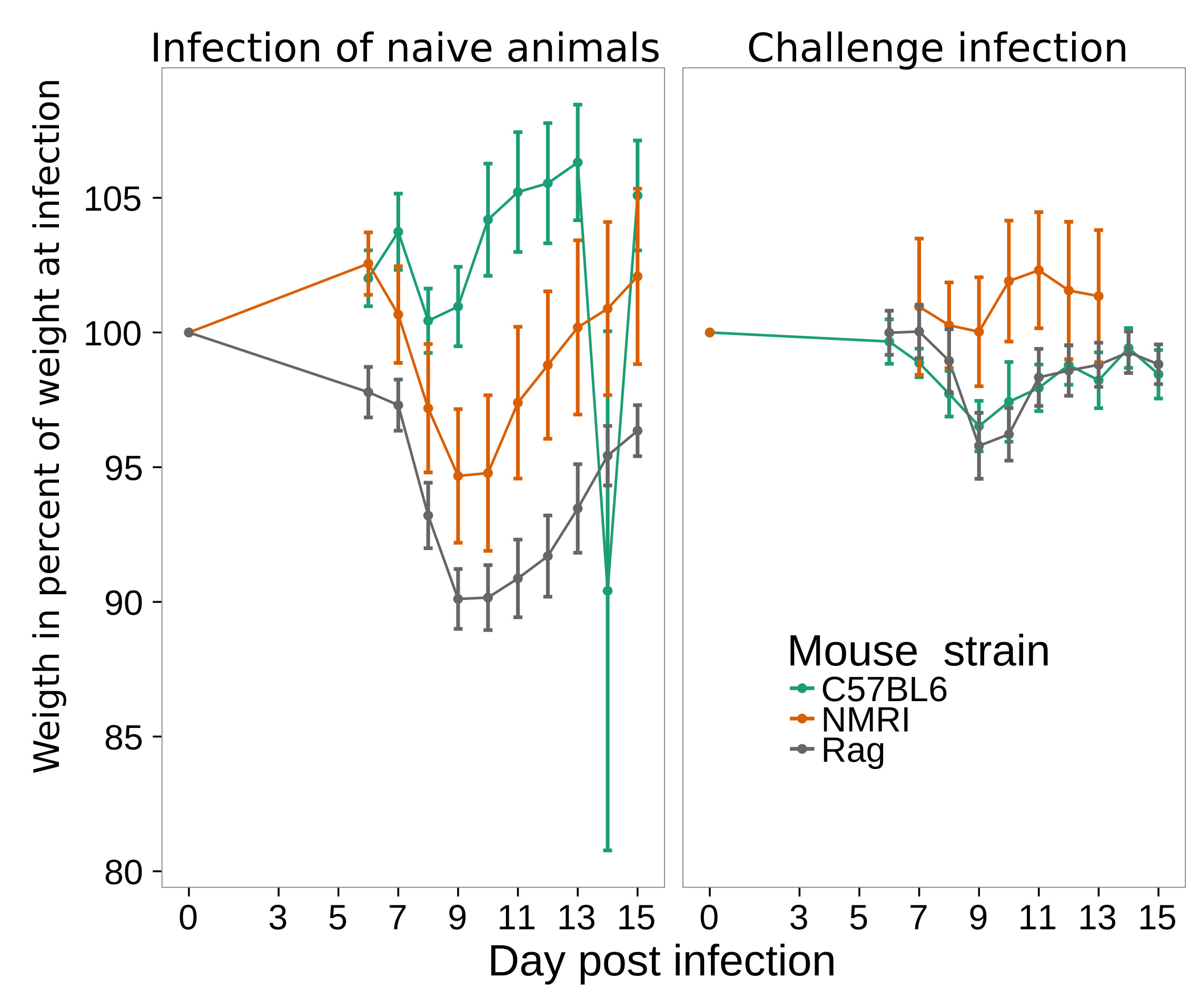

Supplement: Supplementary file 4 — Weight loss of mice during E. falciformis infection. Mouse weight is shown as a percentage relative to weight at the time of infection. Infection dose for NMRI was 150 oocysts in naïve infection and 1500 in challenge infection. For C57BL/6 and Rag1−/− dose was 10 oocysts in both naïve and challenge infection. Bars indicate standard error for three or four replicates. (JPEG 344 kb) [file 12864_2017_4095_MOESM4_ESM.jpg]

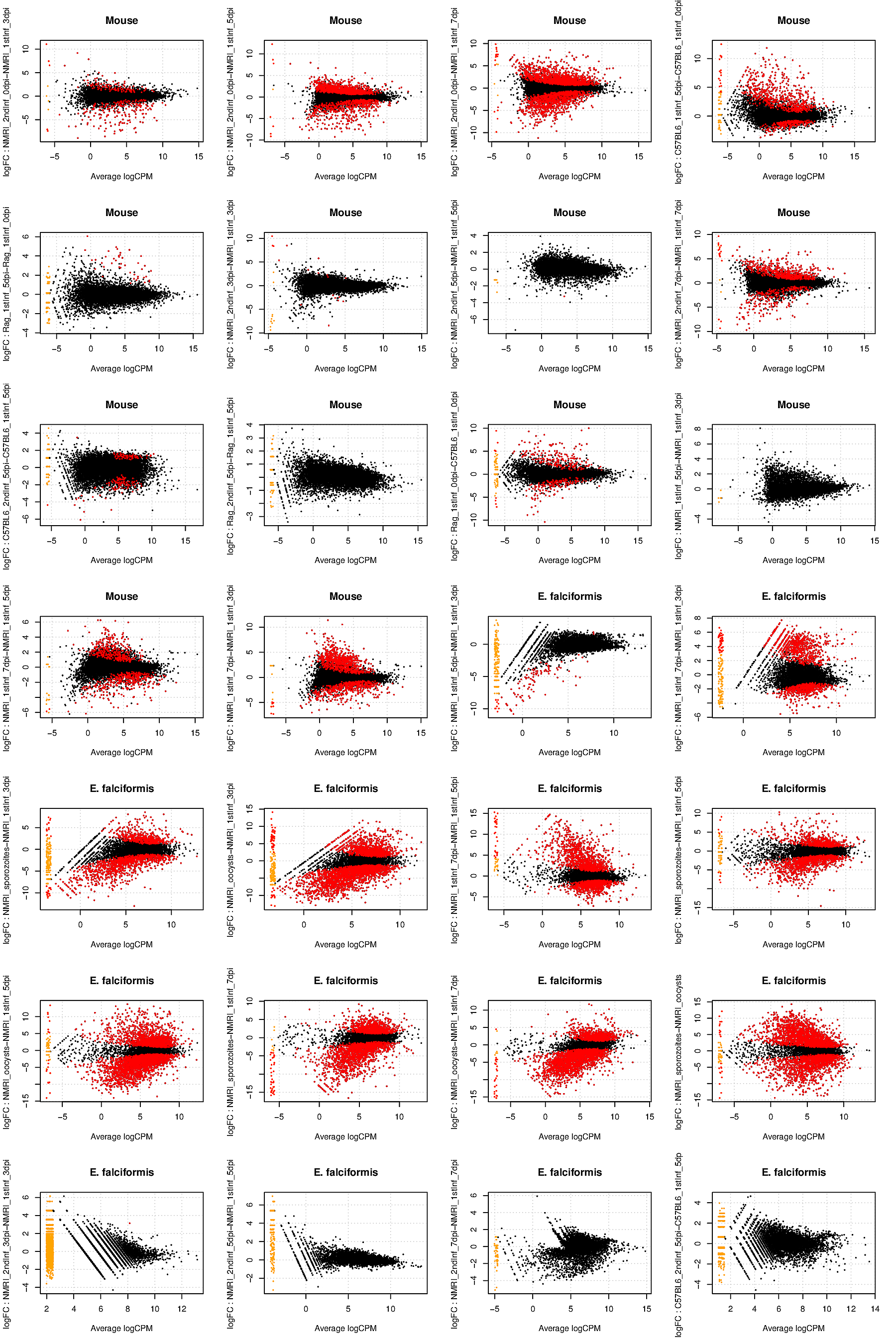

Supplement: Supplementary file 5 — Mean expression level vs. difference in between experimental groups. Mean expression levels are plotted in average counts per million (AverageCPM) vs. the log-Fold change for a particular comparison. Genes showing significant expression differences at a false discovery rate (FDR) threshold <0.01 are highlighted in red. Data for transcripts with zero abundance in one library is indicated using a “smearing” for respective AverageCPM values. (JPEG 2311 kb) [file 12864_2017_4095_MOESM5_ESM.jpg]
